# Glucose-mediated *de novo* lipogenesis in photoreceptors drives early diabetic retinopathy

**DOI:** 10.1016/j.jbc.2021.101104

**Published:** 2021-08-20

**Authors:** Rithwick Rajagopal, Beau Sylvester, Sheng Zhang, Sangeeta Adak, Xiaochao Wei, Megan Bowers, Sebastian Jessberger, Fong-Fu Hsu, Clay F. Semenkovich

**Affiliations:** 1 Department of Ophthalmology and Visual Sciences, Washington University School of Medicine, Saint Louis, Missouri, USA; 2 Division of Endocrinology, Metabolism, and Lipid Research, Washington University School of Medicine, Saint Louis, Missouri, USA; 3 Laboratory of Neural Plasticity, Faculties of Medicine and Science, Brain Research Institute, University of Zurich, Zurich, Switzerland; 4 Department of Cell Biology and Physiology, Washington University School of Medicine, Saint Louis, Missouri, USA

**Keywords:** diabetic retinopathy, lipogenesis, retina, retinal metabolism, membrane lipid, lipotoxicity, fatty acid synthase, ACC, acetyl Co-A carboxylase, DHA, docosahexaenoic acid, DPPC, dipalmitoyl-PC, DR, diabetic retinopathy, ERG, electroretinography, FAS, fatty acid synthase, GFAP, glial fibrillary acidic protein, GOF, gain-of-function, HFD, high-fat diet, IgG, immunoglobulin G, INOS, inducible nitric oxide synthase, LOF, loss of function, OP, oscillatory potential, PC, phosphatidylcholine, PE, phosphatidylethanolamine, SFA, saturated fatty acid, STZ, streptozotocin, VLC-PUFA, very long chain polyunsaturated fatty acid

## Abstract

Diabetic retinopathy (DR) is an increasingly frequent cause of blindness across populations; however, the events that initiate pathophysiology of DR remain elusive. Strong preclinical and clinical evidence suggests that abnormalities in retinal lipid metabolism caused by diabetes may account for the origin of this disease. A major arm of lipid metabolism, *de novo* biosynthesis, is driven by elevation in available glucose, a common thread binding all forms of vision loss in diabetes. Therefore, we hypothesized that aberrant retinal lipid biogenesis is an important promoter of early DR. In murine models, we observed elevations of diabetes-associated retinal *de novo* lipogenesis ∼70% over control levels. This shift was primarily because of activation of fatty acid synthase (FAS), a rate-limiting enzyme in the biogenic pathway. Activation of FAS was driven by canonical glucose-mediated disinhibition of acetyl-CoA carboxylase, a major upstream regulatory enzyme. Mutant mice expressing gain-of-function FAS demonstrated increased vulnerability to DR, whereas those with FAS deletion in rod photoreceptors maintained preserved visual responses upon induction of diabetes. Excess retinal *de novo* lipogenesis—either because of diabetes or because of FAS gain of function—was associated with modestly increased levels of palmitate-containing phosphatidylcholine species in synaptic membranes, a finding with as yet uncertain significance. These findings implicate glucose-dependent increases in photoreceptor *de novo* lipogenesis in the early pathogenesis of DR, although the mechanism of deleterious action of this pathway remains unclear.

Therapy for diabetic retinopathy (DR), a common cause of visual disability, has evolved greatly over the past 2 decades but remains ineffective for up to one-third of patients (1). Development of new strategies for treatment could be facilitated by gaining better insight into the pathophysiology of early stage DR. Abnormalities in retinal lipid metabolism occur early in the course of diabetes and are therefore attractive candidates for mediation of mechanisms that eventually result in vision loss (2).

Retinal lipid abnormalities in diabetes involve both accumulation of deleterious lipids as well as depletion of beneficial ones. Strategies to correct such abnormalities have the potential to lower risk of vision loss from diabetes. For example, reversal of cholesterol accumulation in retinas of streptozotocin (STZ)-induced diabetic mice using liver X receptor agonists reduces severity of DR (3). Similarly, lowering of retinal ceramide levels in diabetes by inhibiting acid sphingomyelinase activity (4) prevents vascular degeneration (5). Complex and very long chain polyunsaturated fatty acids (VLC-PUFA), including docosahexaenoic acid (DHA) and eicosapentaenoic acid, are disproportionately abundant in the retina compared with other tissues (6, 7) and are needed to maintain retinal health but are reduced by diabetes (8, 9). In experimental models, dietary PUFA replacement reduces DR severity (10), and in a randomized clinical trial, DHA supplementation was associated with lower DR severity scores (11).

Photoreceptors, which are increasingly recognized loci of early DR pathogenesis (12), are particularly susceptible to perturbations in membrane lipid composition—specifically because of defects in biosynthetic machinery. For example, reduction of photoreceptor DHA because of targeted disruption of the biosynthetic enzyme lysophosphatidic acid acyltransferase 3 resulted in synaptic dysmorphology and vision loss (13). Diabetes also reduces DHA biosynthesis because of downregulation of elongation of VLC fatty acids protein 4 (ELOVL4), whereas ELOV4 overexpression can counteract some of the effects of diabetes on the retina (14). More broadly, lipid derangements because of disruption of rod photoreceptor fatty acid synthase (FAS)—the enzyme that catalyzes the committed step of *de novo* lipogenesis (15)—causes loss of retinal DHA and VLC-PUFAs, resulting in a rapid neurodegeneration (16). FAS is a multifunctional enzyme made up of eight individual catalytic centers, and it generates palmitate (C16:0) from malonyl-CoA and acetyl-CoA using NADPH as a cofactor. This pathway is essential for multiple arms of cellular lipid biosynthesis, including those responsible for VLC fatty acid production (16).

Diabetes is associated with tissue-specific changes in FAS, which may be root causes of its complications (17, 18). In macrophages, FAS dysfunction in diabetes results in rearrangement of plasma membrane fatty acids, causing impaired or aberrant cellular signaling (19). In skeletal muscle, FAS activity and expression are increased in the setting of insulin resistance, causing increased saturation index of membrane lipids (20). However, the effects of diabetes on this important and elemental lipid biogenesis pathway in the retina are incompletely described. Here, we describe studies examining changes to FAS enzyme activity during diabetes, effects of FAS manipulation on DR phenotypes, and associated changes to the retinal lipid landscape during experimental diabetes.

## Results

### Diabetes is associated with elevated activity of retinal *de novo* lipogenesis

**Figure 1:**
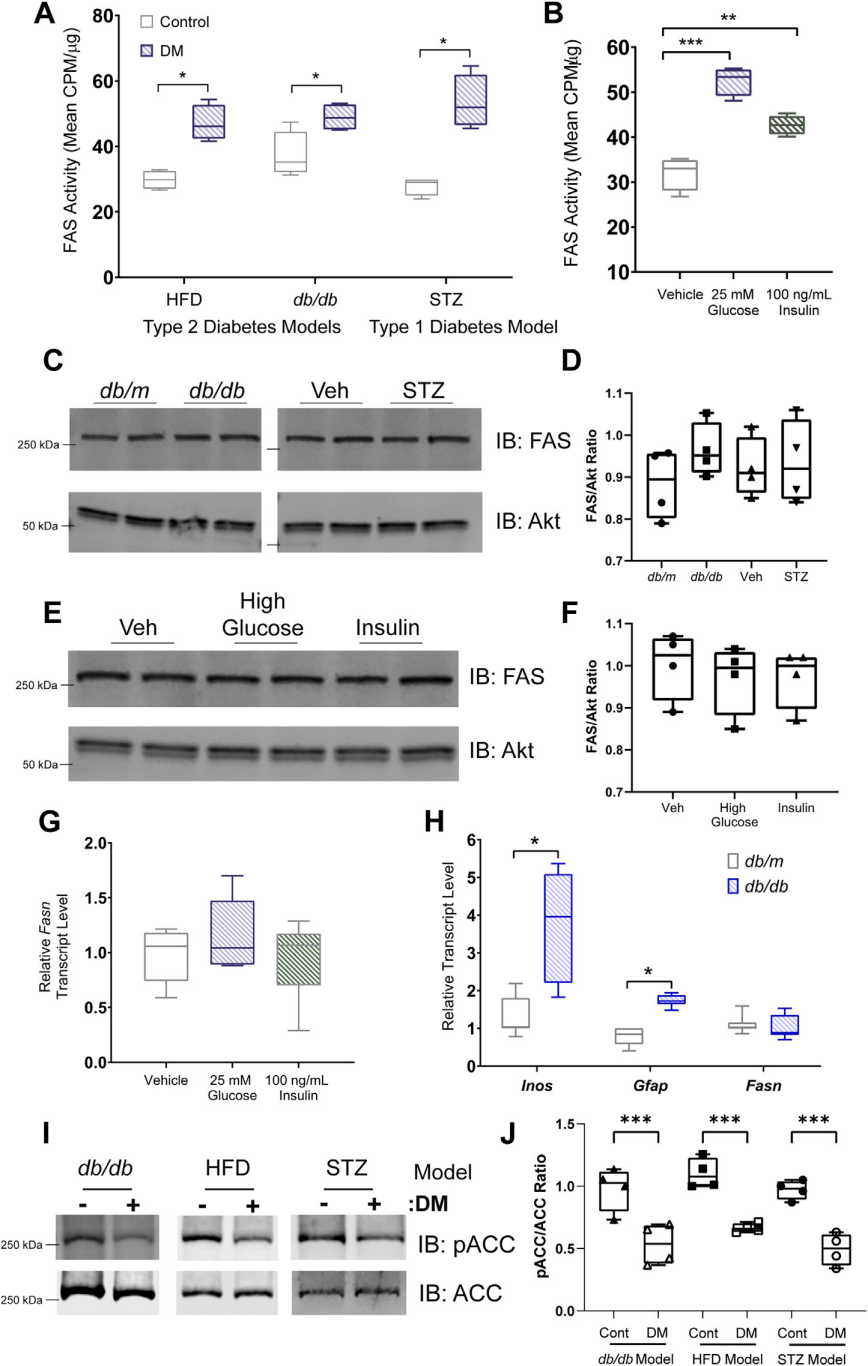
**Increased rate of *de novo* lipogenesis in the diabetic retina.***A*, using a radiolabel incorporation assay that interrogates fatty acid synthase (FAS) function, rates of *de novo* lipogenesis were measured from whole retinal extracts derived from animals after 3 months of exposure to DM in three different models (high-fat diet feeding, HFD; leptin receptor mutants, *db*/*db*; or streptozotocin-induced, STZ) or from healthy littermates. *B*, FAS activity in retinal explants derived from 3-month-old WT animals and cultured for 6 h in control media (5 mM d-glucose + 20 mM l-glucose), high glucose (25 mM d-glucose), or high insulin (5 mM d-glucose + 20 mM l-glucose + 100 ng/ml insulin). *C* and *D*, Western blots and quantification for FAS or a loading control (Akt) from retinal extracts derived from animals with diabetes (*db*/*db*; STZ injected) or their healthy controls (*db*/*m*; vehicle treated). *E* and *F*, Western blots and quantification for FAS or Akt from retinal explant cultures in control, high glucose, or high insulin conditions for 6 h. *G*, quantitative polymerase chain reaction for *Fasn* from retinal explants cultured for 6 h in control media or media containing either high glucose or high insulin. *H*, quantitative polymerase chain reaction from retinal extracts of *db/db* mice or healthy *db/m* controls for the gene targets indicated. *I* and *J*, Western blots and quantification for phosphorylated acetyl co-A carboxylase (ACC) or a total ACC from retinal extracts derived from animals with diabetes (*db*/*db*; HFD; STZ injected) or their healthy controls (*db*/*m*; chow fed; vehicle treated). Data represent mean with interquartile range (*box*) and range (*whiskers*) and were analyzed by Student's *t* test (*A*, *D*, *G*, and *J*) or one-way ANOVA (*B*, *F*, and *H*). ∗*p* < 0.05, ∗∗*p* < 0.01, and ∗∗∗*p* < 0.001. DM, diabetes mellitus.

To investigate whether flux through *de novo* lipogenesis is affected by diabetes, we performed enzyme activity assays in retinal tissues for FAS. We used three independent models of diabetes—leptin receptor–deficient mice (*db/db*), high-fat diet (HFD)–induced disease, and STZ-induced disease (two models of type 2 diabetes and one type 1 model)—that are all associated with progressive retinal dysfunction and dysmorphology (21, 22, 23). After ∼3 months of exposure to diabetes in all models, *de novo* lipogenesis was assessed by measuring the incorporation of radiolabeled malonyl-CoA precursor into palmitate—a reaction uniquely catalyzed by FAS. The controls for these experiments were healthy littermates of the diseased animals (*db/m* as controls for *db/db*, chow-fed mice for the HFD model, and vehicle-injected mice in the STZ model). Across all three models, diabetes was associated with ∼70% increase in retinal FAS activity compared with controls (Fig. 1*A*). Since carbohydrate availability is an important determinant of biosynthetic flux, we next performed *in vitro* assays using short-term retinal explants derived from 3-month-old healthy WT mice to determine the effect of glucose on retinal FAS activity. Exposure of retinal tissue to high glucose (25 mM d-glucose) for 6 h caused an ∼60% increase in FAS activity compared with an osmotically balanced low glucose control medium (5 mM d-glucose + 20 mM l-glucose) (Fig. 1*B*). These increases were similar to the effects of insulin (100 ng/ml), which is known to positively modulate FAS activity through allosteric effects (Fig. 1*B*).

To determine whether diabetes caused elevated FAS activity *via* changes in *Fasn* mRNA or protein levels, we performed Western blotting and quantitative PCR in retinal tissues. FAS protein levels were unchanged between healthy retinas or those exposed to one of three forms of experimental diabetes (Fig. 1, *C* and *D*). Similarly, a 6-h-high glucose pulse or insulin treatment was not associated with changes in FAS protein expression or RNA levels in retinal explants (Fig. 1, *E*–*G*). Whereas *db/db* retinas contained elevated messenger RNA levels of reactive factors, such as inducible nitric oxide synthase (INOS) and glial fibrillary acidic protein (GFAP), they had similar *Fasn* messenger RNA levels as control *db/m* tissue (Fig. 1*H*).

These results suggest that elevated FAS activity in the retina during diabetes is caused by a post-translational mechanism and is responsive to elevated glucose alone. We therefore examined the properties of acetyl Co-A carboxylase (ACC) in the diabetic retina since this enzyme is activated by elevated cellular glucose concentration and acts as an important regulator of FAS activity by regulating the production of its necessary substrate, malonyl-CoA. ACC undergoes inhibitory phosphorylation under conditions of low cellular glucose and conversely becomes dephosphorylated in high glucose concentrations (24). In all three models of experimental diabetes, we found that ACC was hypophosphorylated relative to healthy controls, indicative of increased retinal ACC activity in diabetes (Fig. 1, *I* and *J*).

### Rod photoreceptors are the predominant source of retinal FAS activity

**Figure 2:**
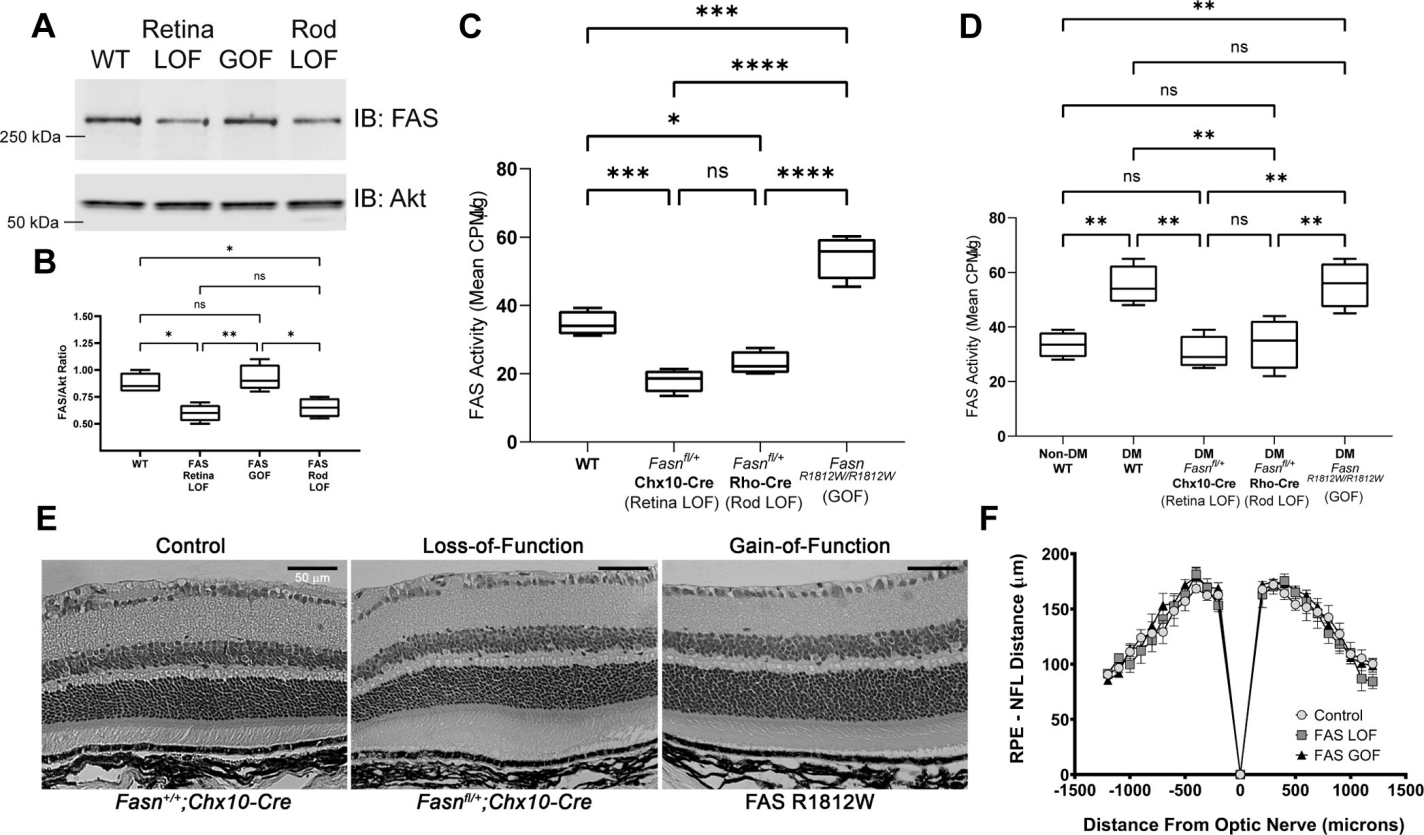
**Manipulation of retinal FAS activity using loss-of-function and gain-of-function approaches.***A* and *B*, Western blots and quantification for FAS from retinal extracts of C57Bl/6J mice (WT), *Fasn*^*fl/+*^;*Chx10*-Cre^*+/−*^ mice (retina LOF), *Fasn*^R1812W/R1812W^ mice (GOF), or *Fasn*^*fl/+*^; *i75*-Cre^*+/−*^ mice (rod LOF). *C*, FAS activity from retinal extracts of WT, retina LOF, rod LOF, or GOF mice. *D*, retinal FAS activity from WT, retina LOF, rod LOF, or GOF mice with 3 months of exposure to high-fat diet–induced DM compared with healthy WT mice (non-DM). *E*, representative hematoxylin and eosin–stained paraffin cross sections of retinas from control, retina LOF, and GOF mice with the indicated genotypes and (*F*) quantification of retinal thicknesses as measured as distances from the nerve fiber layer (NFL) to retinal pigment epithelium (RPE). *B*–*D*, data represent mean with interquartile range (*box*) and range (*whiskers*) and were analyzed by one-way ANOVA. *F*, data represent mean with SEM and were analyzed by two-way ANOVA. ∗*p* < 0.05, ∗∗*p* < 0.01, ∗∗∗*p* < 0.001, and ∗∗∗∗*p* < 0.0001. DM, diabetes mellitus; FAS, fatty acid synthase; GOF, gain of function; LOF, loss of function.

We next characterized mice carrying genetic modifications at the *Fasn* locus causing loss-of-function (LOF) and gain-of-function (GOF) phenotypes. In prior studies, we showed that homozygous deletion of *Fasn* from the neural retina (*Fasn*^*fl/fl*^; Chx10-Cre-driven *loxp* recombination) was associated with a rapid and early onset neurodegeneration, making this reagent unsuitable for a long-term DR modeling experiment (16). Instead, we generated *Fasn*^*fl/+*^*;Chx10-Cre*^*+/−*^ (“retina LOF”), as these heterozygotes do not have any discernible baseline phenotype. These mice demonstrated a ∼50% reduction in FAS protein level in the retina compared with controls, consistent with our prior observations, and a concomitant ∼50% reduction in FAS enzyme activity compared with controls (Fig. 2, *A*–*C*). Since rod photoreceptors make up >80% of the cell population in the mouse retina, we also generated mutants with targeted heterozygous loss of rod FAS using the i75-Cre transgene (*Fasn*^*fl/+*^*;i75-Cre*^*+/−*^; “rod LOF”) (25). These mice showed nearly identical losses in FAS protein level and enzyme activity as retina LOF mice (Fig. 2, *A*–*C*), suggesting that rod photoreceptors are the predominant cellular loci of retinal *de novo* lipogenesis. After induction of diabetes with HFD for 3 months, retina LOF and rod LOF mice have ∼50% less FAS enzyme activity than controls on HFD and are on par with non–diabetes mellitus controls, indicating that both LOF mutants effectively neutralize the retinal FAS activity gains caused by chronic HFD (Fig. 2*D*).

### FAS GOF variant

We also characterized baseline retinal phenotypes of mice with homozygous knock-in of a GOF FAS variant associated with human intellectual disability and impaired adult neurogenesis (26). This variant, which results in a single amino acid substitution of tryptophan for arginine at residue 1812 in mice and 1819 in humans (R1812W), demonstrates a ∼50% increase in enzymatic activity compared with the WT control in brain tissue (27). Similarly, we found that mice carrying this variant have ∼50% elevation in retinal FAS activity compared with controls but with no associated increase in protein expression (Fig. 2, *A*–*C*). Interestingly, the GOF mice showed no additive increase in enzyme activity after induction of diabetes compared with controls (Fig. 2*D*). Even at 12 months of age, we did not observe any degenerative changes or other gross morphological phenotype in retina LOF, rod LOF, or GOF mutants compared with controls (Fig. 2, *E* and *F*).

### Effects of FAS reduction in rods on DR phenotypes

**Figure 3:**
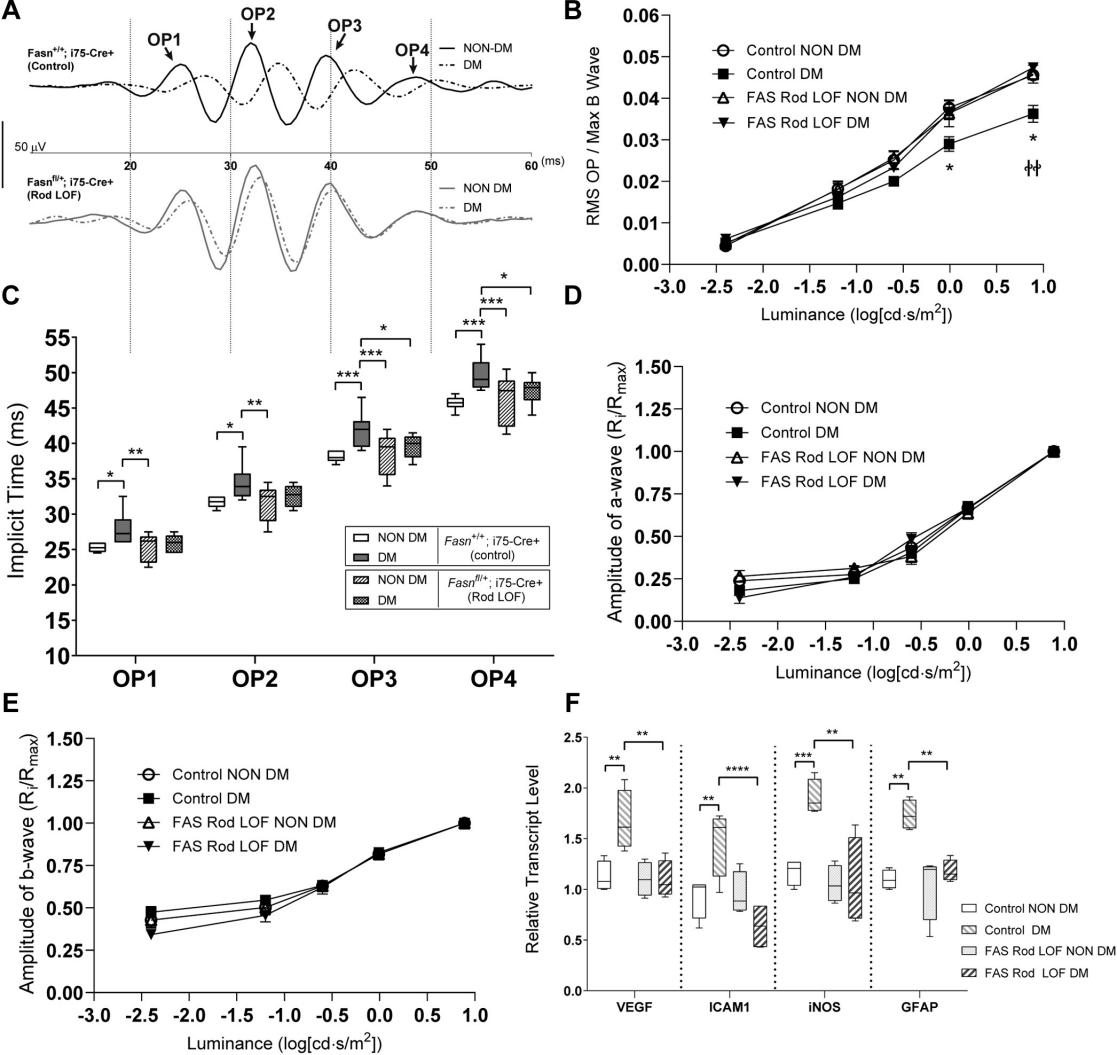
**Effects of FAS haploinsufficiency in rod photoreceptors on diabetes-induced electroretinographic deficits and reactive gene expression.***A*, representative oscillatory potential (OP) waveforms from scotopic electroretinograms after a 0.25 cd·s/m^2^ white flash showing four major peaks (OP 1–4) in control animals (*Fasn*^+/+^; *i75*-Cre^*+/−*^) or rod LOF (*Fasn*^*fl/+*^; *i75*-Cre^*+/−*^) mice that were subjected to high-fat diet (HFD)–induced diabetes mellitus (DM) or standard chow feeding (non-DM). *B*, luminance-response curves of normalized scotopic OP amplitudes in control mice or mice with FAS rod LOF, comparing HFD-induced DM or non-DM (chow-fed) conditions. *C*, scotopic OP implicit times (latencies) after a 0.25 cd·s/m^2^ white flash for each major peak in non-DM mice or those with HFD-induced DM. *D*, scotopic a-wave luminance-response curves and (*E*) b-wave luminance response curves from the indicated groups, represented as normalized ratios of the maximal flash amplitude after a 7.863 cd·s/m^2^ stimulus. *F*, quantitative polymerase chain reaction for the gene targets indicated from retinal extracts of mice after 12 months of exposure to HFD-induced DM or under healthy chow-fed condition (non-DM) controls. *B*, *D*, and *E*, data represent mean with SEM and were analyzed by two-way ANOVA. *C* and *F*, data represent mean with interquartile range (*box*) and range (*whiskers*) and were analyzed by one-way ANOVA. ∗*p* < 0.05, ∗∗*p* < 0.01, ∗∗∗*p* < 0.001, and ∗∗∗∗*p* < 0.0001. In panel *B*, ∗*p* < 0.05 comparing “control NON DM” to “control DM”; ^††^*p* < 0.01 comparing “control DM” to “Fas Rod Het DM.” FAS, fatty acid synthase; LOF, loss of function.

We next assessed whether DR phenotypes are affected by FAS rod LOF mice. After HFD induction, we performed electroretinography (ERG) to assess scotopic oscillatory potential (OP) characteristics—features that are associated with early retinal damage in human diabetes and features that our group has shown to precede the onset of typical vascular lesions in this mouse model (23). Whereas HFD for 6 months causes increased latency of OP timing across all four major peaks and loss of OP amplitudes compared with nondiabetic littermates in control animals, such changes were not seen in mice with FAS rod LOF (Fig. 3, *A*–*C*). Similar to our previous observations, HFD does not impact scotopic a-wave or b-wave amplitudes, and background FAS rod LOF does not change this feature of the ERG (Fig. 3, *D* and *E*). These results argue that diabetes-specific ERG deficits in HFD-fed mice are reversible by partial loss of FAS activity. To confirm that the protective changes extended beyond ERG effects, we performed quantitative PCR for gene products that are known to react to diabetes-induced stress in the retina. Whereas HFD-fed mice had elevated messenger RNA levels of vascular endothelial growth factor-a, intercellular adhesion molecule 1, INOS and GFAP, rod LOF mice on HFD had no such elevations compared with rod LOF on chow diet or compared with control mice on chow diet (Fig. 3*F*).

### Effects of FAS GOF on retinal function in diabetes

**Figure 4:**
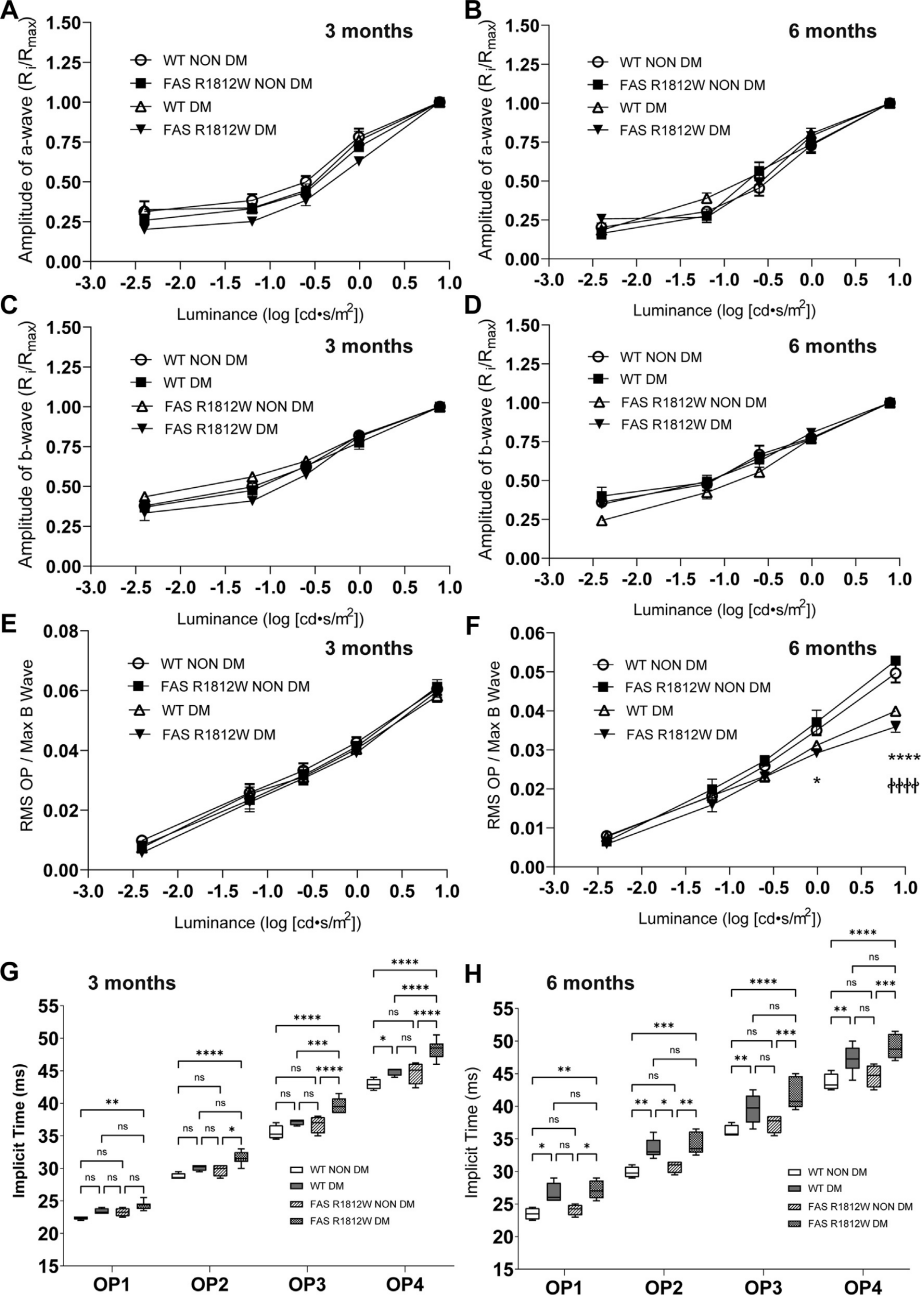
**Effects of FAS gain-of-function on retinal function during the course of high-fat diet–induced diabetes.** Scotopic a-wave luminance-response curves, represented as normalized ratios of the maximal flash amplitude after a 7.863 cd·s/m^2^ stimulus, at 3 months (*A*) and 6 months (*B*) after DM induction in control mice or mice carrying the FAS R1812W gain-of-function (GOF) polymorphism. Normalized b-wave luminance-response curves at 3 months (*C*) and 6 months (*D*) after DM induction in control or GOF mice. Luminance-response curves of normalized scotopic oscillatory potential (OP) amplitudes at 3 months (*E*) and 6 months (*F*) after DM induction in control and GOF mice. Scotopic OP implicit times (latencies) after a 0.25 cd·s/m^2^ white flash for each major peak in non-DM mice or those with HFD-induced DM after 3 months of exposure (*G*) or 6 months of exposure (*H*) to experimental diet, and with or without FAS GOF. *A*–*F*, data represent mean with SEM and were analyzed by two-way ANOVA. *G* and *H*, data represent mean with interquartile range (*box*) and range (*whiskers*) and were analyzed by one-way ANOVA. ∗*p* < 0.05, ∗∗*p* < 0.01, ∗∗∗*p* < 0.001, and ∗∗∗∗*p* < 0.0001. In panel *F*, ∗*p* < 0.05, ∗∗∗∗*p* < 0.0001 comparing “WT NON DM” to “WT DM”; ^††††^*p* < 0.0001 comparing “FAS R1812W NON DM” to “FAS R1812W DM.” DM, diabetes mellitus; FAS, fatty acid synthase.

Using mice homozygous for the FAS R1812W variant, we induced diabetes by HFD and measured retinal function by scotopic ERG. As previously observed, we found that HFD was associated with loss of OP amplitudes, without effects on a-wave or b-wave amplitudes, and only at 6 months of exposure to disease but not at an earlier time point (Fig. 4, *A*–*F*). FAS GOF on HFD showed no changes in this pattern of disease expression and did not impact the severity of OP amplitude loss at 6 months compared with controls (Fig. 4*F*). However, while FAS GOF showed similar delays in OP timing after 6 months of HFD exposure compared with controls, we also observed such delays after 3 months of HFD exposure when control animals had not yet manifested the phenotype (Fig. 4, *G* and *H*). FAS GOF mice on chow diet showed no difference in ERG characteristics compared with WT controls on chow diet at any age tested (up to 12 months). Together, these results suggest that elevated FAS activity alone is insufficient to reproduce the effects of diabetes on retinal function loss, but that it does accelerate such effects.

### Effects of diabetes and elevated FAS activity on retinal synaptic lipids

Our observations with FAS mutants on DR phenotypes in mice suggest that abnormally elevated activity of this enzyme directly contributes to the pathogenesis of vision loss in diabetes. To explore a potential mechanism, we performed lipid surveys of isolated retinal synaptic membranes, as these structures are highly susceptible to changes in retinal FAS activity (16). Among sucrose gradient fractions enriched for retinal synaptic membranes, we observed an increase in phosphatidylcholines (PCs) containing saturated fatty acids (SFAs)—specifically those with C16:0—in diabetic *db/db* mice compared with nondiabetic *db/m* littermate controls (Table 1). In addition, diabetic samples had lower relative content of PCs with unsaturated fatty acids and long alkyl chains (>20 C) compared with nondiabetic controls, consistent with observations performed by others using whole-retina extracts. After performing the same analysis in samples from FAS GOF mice (nondiabetic) and comparing to WT control tissue, we found that excess FAS activity caused a 4% elevation of retinal synaptic dipalmitoyl-PC (DPPC, PC 16:0/16:0) (Table 2). Of note, FAS GOF mice did not have any relative depletion of PUFA-containing PCs and, on the contrary, had elevated levels of PC 22:6/22:6 compared with controls (Table 2). The changes we observed in synaptic membrane content in diabetic mice were specific, since no observed differences were seen among lyso-PCs (Table 3). In FAS GOF, we observed a moderate increase in lyso-PCs 32:6 compared with WT controls (Table 4). We observed no changes in phosphatidylethanolamines (PEs) over controls in tissues from diabetic mice (Table 5) or FAS GOF mice (Table 6).

**Table 1:** Changes in normalized retinal synaptic membrane PC content associated with experimental diabetes

PC species	Control (95% confidence interval); n = 6	Diabetes (95% confidence interval); n = 6	*p*
14:0/16:1	0.061 (0.057–0.065)	0.069 (0.067–0.071)	0.004
14:0/16:0	0.043 (0.040–0.046)	0.041 (0.039–0.044)	0.425
p16:0/16:1	0.006 (0.005–0.007)	0.007 (0.007–0.008)	0.012
a16:0/16:1	0.018 (0.016–0.019)	0.022 (0.021–0.024)	0.002
16:1/16:1	0.011 (0.010–0.013)	0.014 (0.013–0.015)	0.007
16:0/16:1	0.157 (0.152–0.162)	0.163 (0.159–0.167)	0.109
16:0/16:0	1.012 (0.981–1.043)	1.015 (0.982–1.048)	0.896
p16:0/18:1	0.026 (0.024–0.028)	0.034 (0.032–0.037)	0.0006
a16:0/18:1	0.022 (0.022–0.023)	0.022 (0.021–0.023)	0.996
16:1/18:2	0.012 (0.011–0.013)	0.015 (0.013–0.016)	0.009
16:0/18:2; 16:1/18:1	0.104 (0.098–0.109)	0.124 (0.113–0.134)	0.007
16:0/18:1	0.354 (0.348–0.360)	0.356 (0.347–0.365)	0.764
p16:0/20:4	0.020 (0.019–0.022)	0.022 (0.020–0.023)	0.401
a16:0/20:4	0.016 (0.015–0.018)	0.016 (0.014–0.018)	0.771
a18:0/18:1	0.019 (0.017–0.020)	0.017 (0.015–0.019)	0.166
16:0/20:4	0.109 (0.103–0.116)	0.102 (0.099–0.105)	0.075
16:0/20:3; 18:1/18:2	0.055 (0.053–0.057)	0.058 (0.054–0.062)	0.187
18:1/18:1; 18:0/18:2	0.128 (0.124–0.131)	0.122 (0.119–0.125)	0.030
16:0/22:6	0.509 (0.485–0.532)	0.472 (0.459–0.486)	0.025
18:0/18:1	0.427 (0.395–0.458)	0.428 (0.423–0.432)	0.953
p18:0/20:4	0.014 (0.013–0.016)	0.015 (0.014–0.015)	0.621
a18:0/20:4	0.012 (0.010–0.014)	0.013 (0.012–0.014)	0.746
18:1/20:4; 16:0/22:5	0.102 (0.099–0.106)	0.097 (0.095–0.099)	0.046
18:0/20:4	0.163 (0.149–0.176)	0.149 (0.145–0.153)	0.092
18:1/22:6	0.061 (0.059–0.062)	0.061 (0.058–0.065)	0.771
18:0/22:6	0.658 (0.594–0.721)	0.666 (0.638–0.694)	0.809
18:0/22:5	0.103 (0.094–0.113)	0.106 (0.102–0.110)	0.653
18:0/22:4	0.335 (0.305–0.364)	0.335 (0.322–0.348)	0.977
42:10	0.174 (0.163–0.185)	0.178 (0.167–0.189)	0.642
42:4	0.008 (0.007–0.009)	0.006 (0.005–0.007)	0.026
22:6/22:6	0.151 (0.145–0.157)	0.153 (0.137–0.168)	0.856
22:6/32:6	0.027 (0.025–0.028)	0.023 (0.020–0.026)	0.050
22:6/34:6	0.027 (0.025–0.029)	0.021 (0.018–0.025)	0.019
22:5/24:6	0.018 (0.016–0.020)	0.014 (0.012–0.015)	0.006
22:6/34:6	0.003 (0.003–0.004)	0.002 (0.002–0.003)	0.003

Synaptic membranes isolated by sucrose gradient were analyzed by electrospray ionization mass spectrometry. Identified peaks were normalized to input protein content and to an internal standard. Mice were either 6-month-old *db/db* (diabetes) or littermate *db/m* (control). Groups were compared by two-tailed homoscedastic *t* test.

**Table 2:** Changes in normalized retinal synaptic membrane PC content associated with FAS GOF

PC species	Control (95% confidence interval); n = 6	FAS GOF (95% confidence interval); n = 6	*p*
14:0/16:1	0.067 (0.060–0.074)	0.060 (0.057–0.064)	0.129
14:0/16:0	0.039 (0.037–0.041)	0.042 (0.041–0.043)	0.062
p16:0/16:1	0.007 (0.007–0.008)	0.006 (0.006–0.007)	0.062
a16:0/16:1	0.021 (0.019–0.022)	0.020 (0.019–0.021)	0.514
16:1/16:1	0.011 (0.010–0.013)	0.010 (0.008–0.012)	0.171
16:0/16:1	0.155 (0.149–0.162)	0.145 (0.139–0.151)	0.039
16:0/16:0	0.957 (0.946–0.969)	0.998 (0.988–1.010)	0.0003
p16:0/18:1	0.036 (0.033–0.038)	0.033 (0.031–0.035)	0.135
a16:0/18:1	0.023 (0.022–0.025)	0.022 (0.020–0.023)	0.153
16:1/18:2	0.011 (0.010–0.013)	0.011 (0.010–0.012)	0.858
16:0/18:2; 16:1/18:1	0.097 (0.092–0.010)	0.097 (0.092–0.102)	0.994
16:0/18:1	0.345 (0.339–0.351)	0.355 (0.348–0.361)	0.052
p16:0/20:4	0.017 (0.015–0.019)	0.016 (0.015–0.017)	0.351
a16:0/20:4	0.014 (0.012–0.016)	0.014 (0.013–0.015)	0.860
a18:0/18:1	0.017 (0.015–0.018)	0.017 (0.015–0.019)	0.648
16:0/20:4	0.107 (0.097–0.117)	0.093 (0.090–0.097)	0.025
16:0/20:3; 18:1/18:2	0.053 (0.051–0.055)	0.052 (0.050–0.055)	0.529
18:1/18:1; 18:0/18:2	0.113 (0.110–0.116)	0.108 (0.104–0.113)	0.113
p18:0/20:4	0.013 (0.011–0.015)	0.011 (0.010–0.012)	0.104
a18:0/20:4	0.011 (0.009–0.013)	0.011 (0.009–0.013)	0.906
16:0/22:6	0.449 (0.437–0.461)	0.446 (0.418–0.473)	0.827
18:0/18:1	0.414 (0.410–0.419)	0.399 (0.387–0.411)	0.043
18:1/20:4;16:0/22:5	0.091 (0.088–0.094)	0.087 (0.083–0.091)	0.089
18:0/20:4	0.161 (0.149–0.173)	0.142 (0.137–0.147)	0.018
18:1/22:6	0.052 (0.051–0.054)	0.054 (0.052–0.056)	0.114
18:0/22:6	0.583 (0.566–0.600)	0.576 (0.547–0.605)	0.690
18:0/22:5	0.095 (0.092–0.097)	0.090 (0.086–0.095)	0.146
40:4	0.030 (0.029–0.031)	0.029 (0.028–0.030)	0.032
42:10	0.015 (0.014–0.017)	0.013 (0.011–0.015)	0.051
42:4	0.007 (0.007–0.008)	0.007 (0.005–0.008)	0.233
22:6/22:6	0.087 (0.081–0.092)	0.100 (0.094–0.105)	0.006
22:6/32:6	0.018 (0.017–0.019)	0.017 (0.015–0.018)	0.255
22:6/34:6	0.020 (0.018–0.022)	0.018 (0.015–0.021)	0.232
22:5/34:6	0.013 (0.012–0.014)	0.012 (0.009–0.014)	0.314
22:6/36:6	0.003 (0.002–0.003)	0.003 (0.002–0.003)	0.523

Synaptic membranes isolated by sucrose gradient were analyzed by electrospray ionization mass spectrometry. Identified peaks were normalized to input protein content and to an internal standard. Mice were either 6-month-old *Fasn*^*+/+*^ (control) or age-matched *Fasn*^*R1812W/R1812W*^ (FAS GOF). Groups were compared by two-tailed homoscedastic *t* test.

**Table 3:** Changes in normalized retinal synaptic membrane lyso-PC content associated with experimental diabetes

Lyso-PC species	Control (95% confidence interval); n = 6	Diabetes (95% confidence interval); n = 6	*p*
16:0	0.296 (0.221–0.371)	0.850 (−0.216 to 1.916)	0.333
18:1	0.139 (0.103–0.176)	0.391 (−0.085 to 0.867)	0.325
18:0	0.137 (0.106–0.167)	0.392 (−0.087 to 0.870)	0.322
20:0	0.027 (0.021–0.034)	0.075 (−0.019 to 0.169)	0.345
22:6	0.190 (0.144–0.236)	0.538 (−0.131 to 1.207)	0.333
32:6	0.026 (0.024–0.029)	0.071 (−0.017 to 0.159)	0.344

Synaptic membranes isolated by sucrose gradient were analyzed by electrospray ionization mass spectrometry. Identified peaks were normalized to input protein content and to an internal standard. Mice were either 6-month-old *db/db* (diabetes) or littermate *db/m* (control). Groups were compared by two-tailed homoscedastic *t* test.

**Table 4:** Changes in normalized retinal synaptic membrane lyso-PC content associated with FAS GOF

Lyso-PC species	Control (95% confidence interval); n = 6	FAS GOF (95% confidence interval); n = 6	*p*
16:0	2.747 (2.178–3.315)	2.807 (2.359–3.255)	0.874
18:1	1.384 (1.098–1.669)	1.300 (1.089–1.511)	0.655
18:0	1.272 (1.003–1.541)	1.380 (1.124–1.635)	0.582
20:0	0.272 (0.216–0.328)	0.226 (0.193–0.259)	0.202
22:6	1.494 (1.197–1.792)	1.487 (1.239–1.736)	0.973
32:6	0.181 (0.100–0.262)	0.349 (0.227–0.470)	0.048

Synaptic membranes isolated by sucrose gradient were analyzed by electrospray ionization mass spectrometry. Identified peaks were normalized to input protein content and to an internal standard. Mice were either 6-month-old *Fasn*^*+/+*^ (control) or age-matched *Fasn*^*R1812W/R1812W*^ (FAS GOF). Groups were compared by two-tailed homoscedastic *t* test.

**Table 5:** Changes in normalized retinal synaptic membrane PE content associated with experimental diabetes

PE species	Control (95% confidence interval); n = 6	Diabetes (95% confidence interval); n = 6	*p*
16:0/16:1	0.017 (0.011–0.023)	0.022 (0.003–0.040)	0.608
16:0/18:2	0.014 (0.005–0.022)	0.012 (0.008–0.016)	0.711
16:0/18:1	0.034 (0.024–0.044)	0.068 (0.055–0.081)	0.002
p16:0/20:4	0.248 (0.176–0.321)	0.338 (0.296–0.381)	0.062
16:0/20:4	0.052 (0.040–0.064)	0.052 (0.036–0.069)	0.984
18:1/18:1	0.032 (0.023–0.042)	0.039 (0.024–0.053)	0.487
18:0/18:1	0.128 (0.090–0.166)	0.099 (0.080–0.118)	0.207
p16:0/22:6	0.399 (0.314–0.483)	0.421 (0.359–0.484)	0.684
p16:0/22:5	0.152 (0.122–0.183)	0.206 (0.179–0.232)	0.026
p18:0/20:4	0.275 (0.227–0.323)	0.420 (0.341–0.498)	0.011
16:0/22:6	0.705 (0.647–0.764)	0.759 (0.637–0.881)	0.456
18:1/20:4	0.149 (0.120–0.177)	0.195 (0.152–0.237)	0.109
18:0/20:3	0.107 (0.082–0.131)	0.067 (0.040–0.094)	0.058
p18:0/22:6	0.525 (0.411–0.639)	0.684 (0.631–0.737)	0.032
18:1/22:6	0.199 (0.166–0.231)	0.238 (0.194–0.281)	0.190
18:0/22:4	0.147 (0.111–0.183)	0.192 (0.139–0.244)	0.197

Synaptic membranes isolated by sucrose gradient were analyzed by electrospray ionization mass spectrometry. Identified peaks were normalized to input protein content and to an internal standard. Mice were either 6-month-old *db/db* (diabetes) or littermate *db/m* (control). Groups were compared by two-tailed homoscedastic *t* test.

**Table 6:** Changes in normalized retinal synaptic membrane PE content associated with FAS GOF

PE species	Control (95% confidence interval); n = 6	FAS GOF (95% confidence interval); n = 6	*p*
16:0/16:1	0.038 (0–0.077)	0.019 (0.008–0.029)	0.356
16:0/18:2	0.019 (−0.005 to 0.042)	0.020 (0.008–0.032)	0.915
16:0/18:1	0.105 (−0.029 to 0.238)	0.057 (0.037–0.076)	0.501
p16:0/20:4	0.213 (0.131–0.295)	0.248 (0.192–0.303)	0.506
16:0/20:4	0.037 (0.005–0.068)	0.050 (0.024–0.076)	0.538
18:1/18:1	0.084 (−0.046 to 0.214)	0.016 (0.011–0.022)	0.331
18:0/18:1	0.071 (0.011–0.131)	0.072 (0.046–0.098)	0.970
p16:0/22:6	0.361 (0.218–0.504)	0.336 (0.281–0.391)	0.753
p16:0/22:5	0.144 (0.069–0.219)	0.182 (0.131–0.234)	0.425
p18:0/20:4	0.327 (0.173–0.261)	0.261 (0.196–0.326)	0.455
16:0/22:6	0.583 (0.415–0.751)	0.608 (0.453–0.762)	0.837
18:1/20:4	0.106 (0.086–0.126)	0.197 (0.114–0.279)	0.062
18:0/20:3	0.116 (0.076–0.156)	0.130 (0.090–0.171)	0.633
p18:0/22:6	0.497 (0.265–0.728)	0.546 (0.421–0.671)	0.721
18:1/22:6	0.166 (0.113–0.220)	0.152 (0.137–0.168)	0.631
18:0/22:4	0.210 (0.084–0.337)	0.135 (0.086–0.183)	0.302

Synaptic membranes isolated by sucrose gradient were analyzed by electrospray ionization mass spectrometry. Identified peaks were normalized to input protein content and to an internal standard. Mice were either 6-month-old *Fasn*^*+/+*^ (control) or age-matched *Fasn*^*R1812W/R1812W*^ (FAS GOF). Groups were compared by two-tailed homoscedastic *t* test.

## Discussion

Vision loss from DR commonly occurs because of a progressive vasculopathy that has well-defined ophthalmoscopic characteristics (28). However, damage to the retina in diabetes occurs long before the onset of such lesions and likely involves numerous nonvascular cell types of the retina, notably photoreceptors. In preclinical models, inactivating mutations in components of the rod visual cycle (including *Rho*, *RPE65*, and *Gnat1*), pharmacologic blockade of RPE65, or prolonged visual sensory deprivation are all associated with reduction in DR severity or even outright prevention of any detectable DR (29, 30, 31, 32). Taken together with the long-recognized unique metabolic demands of photoreceptors (33), these observations strongly implicate perturbations in photoreceptor and/or visual cycle metabolism as a key initiating event in the pathogenesis of DR. The current study provides insight into a putative role for an arm of photoreceptor metabolism—that of *de novo* lipogenesis regulated by FAS—as one such molecular pathway altered by diabetes. In type 1 and type 2 models of diabetes, elevated retinal FAS activity was associated with hyperglycemia and disinhibition of ACC, a proximal regulatory enzyme. Nearly, all the excess *de novo* lipogenic activities in the diabetic retina were attributed to rod FAS, whereas targeted loss of FAS in rods mitigated DR-associated scotopic OP amplitude reductions and implicit time delays. Furthermore, we linked elevated FAS activity to increased palmitate content within retinal synaptic membranes, similar to changes seen with diabetes alone.

Our observations are consistent with those of others studying lipid dysmetabolism in the diabetic retina, but some important distinctions deserve attention. Similar to Busik *et al.*, we found that retinal membranes from diabetic mice were relatively depleted of DHA-containing phospholipids (Table 1). In their studies, retinal DHA reductions in diabetes were linked to lowered expression of ELOVL2 and ELOVL4, although the mechanism for suppressed expression of these factors remains unclear (9). Unlike these investigators, we found that several SFA-containing species—particularly those with palmitate (C16:0), such as DPPC—were elevated in DR, albeit modestly. Whereas prior studies examined changes in lipid landscapes because of diabetes in whole-retina extracts, we examined isolated retinal synaptic membranes. This key difference in methodology likely accounts for the disparity between our observations compared with a prior study that did not find changes in SFA content in DR (9).

We chose to study lipid compositions in retinal synaptic membranes for two reasons. First, in our earlier studies of *de novo* lipogenesis in the retina, we found that retinal synaptic membranes were particularly affected by FAS depletion, which was associated with reductions in synaptic SFAs and loss of ribbon architecture (16). In mice with homozygous deletion of *Fasn*, we did not observe a depletion of DPPC in whole-retina extracts. However, after repeating the analysis in gradient fractions enriched for synaptic membranes, we found marked reductions in DPPC in animals lacking retinal FAS. Consistent with these earlier findings, we now found that FAS GOF mice had elevated levels of DPPC within retinal synapses (Table 2). Second, our current findings argue that FAS enzyme changes in diabetes affect ERG function early in the course of disease—pointing to a potential impact on retinal synaptic health. This possibility is feasible since lipid content determines membrane biophysical characteristics, including effects on fluidity and deformability, which can strongly influence synaptic function (34). However, because the lipid changes we observed in synapses were modest, other changes to the retinal lipid environment—such as alterations in retinal triglyceride content or to lipid droplet formation in the outer retina—deserve further study as putative pathogenic mechanisms in FAS GOF mice.

**Figure 5:**
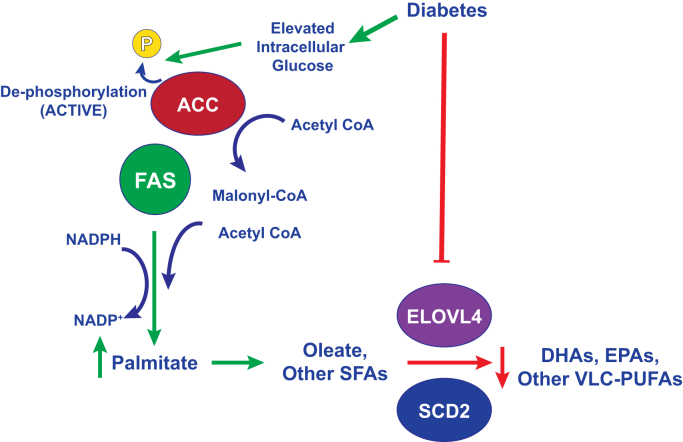
**A model for FAS activity in photoreceptors in early diabetes.** Diabetes-induced increases in photoreceptor glucose concentration result in dephosphorylation (disinhibition) of acetyl-CoA carboxylase (ACC), which in turn increases production of malonyl-CoA—a substrate and allosteric activator of FAS. FAS-mediated production of palmitate promotes subsequent enzymatic reactions that are responsible for production of longer chain saturated fatty acids (SFAs) and unsaturated fatty acids. Simultaneous changes in diabetes, such as reduction in docosahexaenoic acid (DHA), eicosapentaenoic acid (EPA), and other very-long chain polyunsaturated fatty acids (VLC-PUFAs), mediated by suppressed expression of factors such as elongation of VLC fatty acids protein 4 (ELOVL4) and stearyl-CoA desaturase 2 (SCD2), may work in concert to produce membrane changes in the retina to cause pathology in diabetes. FAS, fatty acid synthase.

Importantly, we did not observe a DR-related phenotype (or any structural or functional phenotype) in FAS GOF mice in the absence of background diabetes. This result suggests that overactivity of FAS (on the order of 50% elevation) is insufficient to produce ERG changes related to DR. Instead, we found that scotopic OP delays occurred in an accelerated fashion in FAS GOF mice on HFD compared with WT mice on HFD (3 months *versus* 6 months) (Fig. 4). One explanation accounting for these findings is that elevated retinal enzyme activity in FAS GOF mice is not high enough to reproduce the effect of diabetes. Another explanation is that FAS activity increases are one of many diabetes-associated changes in photoreceptors that initiate early DR. Such a model would account for our finding that retinas from FAS GOF mice had elevated levels of VLC-PUFA (PC-22:6/22:6 and lyso-PC-32:6, Tables 2 and 4) compared with WT controls, in contrast with tissues from diabetic mice, which showed relative loss of VLC-PUFA compared with nondiabetic controls (Table 1). We propose that combinatorial effects of SFA accumulation because of FAS overactivity with other retinal membrane modifications in diabetes, such as loss of VLC-PUFA because of decreased ELOVL4 or stearyl-CoA desaturase activity, could cause the necessary lipid pathology needed to initiate DR (Fig. 5).

Although the evidence for a photoreceptor locus of early pathology in DR is compelling—including some evidence from patients with inherited retinal degeneration from photoreceptor-inactivating polymorphisms—the link between such changes and the eventual development of typical vascular lesions in DR are unclear (35, 36, 37). Data from Busik *et al.* suggest that the loss of VLC-PUFA in diabetes may not be at the level of photoreceptors—even though photoreceptor outer segments are known to have the highest concentration of these lipids. Instead, they propose that endothelial PUFA loss, causing changes in biomechanics of membranes in the vicinity of claudin-5 complexes, could compromise tight-junction physiology and lead to barrier dysfunction (14). Another possibility is that diabetes-induced lipid perturbations at photoreceptor synapses directly impact neurovascular coupling (38). These effects could be mediated by changes in synaptic membrane fluidity and subsequent effects on neurotransmission (34). Also possible is that excess retinal SFA could have deleterious consequences on photoreceptor energy metabolism, which could in turn impact vascular coupling responses. For example, in dorsal root ganglia cultures, lipotoxicity because of excess SFAs—palmitate in particular—contributes to neural dysfunction through mitochondrial damage (39). Alternatively, membrane lipid changes could directly impact second messenger signaling, for example, through changes in arachidonic acids or epoxyeicosatrienoic acids, which are known to mediate neurovascular crosstalk in the retina (40). Studies designed to monitor small-scale vascular caliber changes reproducibly show that experimental and clinical diabetes reduces light-stimulated vasodilation in the retina. Lipogenic pathways impacted by diabetes may account for such changes by directly modifying the metabolic demands and signaling characteristics of photoreceptors. Exploring this possibility could identify a novel target for early therapeutic intervention in DR.

## Experimental procedures

### Animals

C57BL/6J mice were obtained from Jackson Laboratories (stock no. 000664) and were free of rd1 or rd8 mutations. Leptin receptor mutation-carrying BKS.Cg-*Dock7*^*m*^ +/+ *Lepr*^*db/J*^ breeding pairs (*db/m* heterozygotes) were also obtained from Jackson Laboratories (stock no. 000642). F1 progeny were generated with the following expected ratios: 25% homozygous *db/db* animals, which are spontaneously diabetic; 50% *db/m* heterozygous littermates, which are metabolically healthy; and 25% homozygous *m/m* mice, which were routinely discarded. Animals were fed Purina 4043 “chow” (13% kcal from fat, 62% kcal from carbohydrate, and 25% kcal from protein) or Harlan Teklad TD 88137 “HFD” (42% kcal from fat, 43% kcal from carbohydrate, and 15% kcal from protein).

In some experiments, STZ was used to induce diabetes in male mice only, given the known resistance of female C57BL/6J mice to this drug. To do so, 2-month-old mice were fasted for 4 h and then given intraperitoneal doses of STZ, 60 mg/kg dissolved in freshly prepared sodium citrate buffer (pH 4.5) or citrate buffer alone for controls. The procedure was repeated every 24 h for a total of five doses. Random blood glucose was assessed weekly by a portable Glucocard test-strip device (Arkray USA, Inc) using a drop of blood from tail-vein puncture, beginning 1 week after STZ dosing. Animals with glucose values consistently above 300 mg/dl were considered “diabetic.” Weekly body weight was also monitored, and animals with >10% reduction in body weight compared with pre-STZ baseline measurements received regular insulin therapy (2.5 IU/kg in 0.9% NaCl by intraperitoneal administration twice a day) to prevent excess catabolism.

### Antibodies and quantitative PCR

For Western blotting, we used polyclonal rabbit immunoglobulin G (IgG) against FAS (catalog no. ab22759; AbCam), rabbit IgG against ACC phosphorylated at Ser79 (catalog no. 3661; Cell Signaling), rabbit IgG against total ACC (catalog no. 3662; Cell Signaling), and mouse monoclonal IgG1 against pan-Akt (catalog no. 2920; Cell Signaling).

At the indicated ages, total RNA was extracted from isolated retinas. After reverse transcription from 0.5 μg of input, quantitative PCR was performed on the resulting complementary DNA using the following primer pairs.

*Fasn* sense 5′-GTCGTCTGCCTCCAGAGC-3′; *Fasn* antisense 5′-GTTGGCCCAGAACTCCTGTA-3′

Vascular endothelial growth factor-a: sense 5′-AATGCTTTCTCCGCTCTGAA-3′; antisense 5′-GCTTCCTACAGCACAGCAGA-3′

Intercellular adhesion molecule 1: sense 5′-AACAGTTCACCTGCACGGAC-3′; antisense 5′-GTCACCGTTGTGATCCCTG-3′

*Inos*: sense 5′-TGAAGAAAACCCCTTGTGCT-3′; antisense 5′-TTCTGTGCTGTCCCAGTGAG-3′

*Gfap*: sense 5′-TTTCTCGGATCTGGAGGTTG-3′; antisense 5′-AGATCGCCACCTACAGGAAA-3′

*Gapdh*: sense 5′-TGCACCACCAACTGCTTAGC-3′; antisense 5′-GGCATGGACTGTGGTCATGAG-3′

*Rpl32*: sense 5′-GGCTTTTCGGTTCTTAGAGGA-3′; antisense 5′-TTCCTGGTCCACAATGTCAA-3′

Gene expression data were normalized to the mean of two internal controls—Gapdh and Rpl32.

### FAS enzyme activity

Two isolated retinas per animal were homogenized in lysis buffer and centrifuged as aforementioned. One hundred microliters of supernatant was added to a buffer containing 0.1 M potassium phosphate buffer (pH 7.0), 1 mM acetyl-CoA, 1 mM cold malonyl-CoA, 0.1 μCi 14C-malonyl-CoA (catalog no. NEC612005UC; PerkinElmer), 1 mM dithiothreitol, 1 mM EDTA, and 0.5 mM freshly prepared NADPH. This mixture was incubated in a 37 °C agitating bath for 15 min, and reactions were then terminated using 7.5 μl of 60% perchloric acid with immediate vortexing. The resulting cloudy mixture was treated with 0.25 ml of absolute ethanol. Then, 0.75 ml petroleum ether was added, and the mixture was agitated and centrifuged to generate an organic phase containing long-chain fatty acids. Three such extractions with petroleum ether were performed, and the pooled organic phase was added to a scintillation counter to quantitate the radiolabel.

### Retinal morphologic assays

Eyes from male and female mice were fixed in 4% paraformaldehyde at 4 °C for 48 h, paraffin embedded, sectioned into four micron slices, and stained with hematoxylin and eosin.

### ERG

A UTAS BigShot System (LKC Technologies, Inc) was used. Mice (>5 for each group) were dark-adapted overnight. Under red-light illumination, animals were anesthetized with ketamine (80 mg/kg total body weight) and xylazine (15 mg/kg of lean body weight). Pupils were dilated with 1% atropine sulfate; body temperature was maintained at 37 °C with a heating pad; and contact lens electrodes were placed on both eyes along with appropriate reference and ground electrodes. The stimulus was a 10-μs full-field white-light flash. Repeated trials were averaged for each luminance, with ten repeats used for the dimmest flashes and five repeats for the brightest flashes. Raw data were processed using a MATLAB program (MathWorks). The amplitude of the a-wave was measured from the average pretrial baseline to the most negative point of the average trace, and the b-wave amplitude was measured from that point to the highest positive point. The log luminance of the stimulus (log[cd·s/m^2^]) was calculated based on manufacturer's calibrations. OPs were isolated using a digital Butterworth 100 Hz high-pass filter, and time to peak for each OP (OP1–OP4) was determined.

### Lipid analyses by electrospray ionization mass spectrometry

Two retinas from each animal were freshly isolated, pooled into 500 μl of 40% methanol, and homogenized in a glass tube with a Dounce homogenizer. An aliquot of each homogenate (50 μl) was reserved and diluted in deionized water to 10% methanol for total protein measurement using Bradford reagent. Using the remaining homogenate, an appropriate amount of 14:0/14:0-PC and 14:0/14:0-PE internal standard was added before Folch extraction. After extraction, the organic layer was collected, dried under nitrogen, and reconstituted in 200 μl chloroform/methanol (1:1) with 0.1% NH_4_OH. A 10-μl aliquot was loop injected into a Thermo Vantage triple quadrupole mass spectrometer using an Accela autosampler with 1250 HPLC pump, which delivered a constant flow of 40 μl/min of methanol with 0.1% NH_4_OH. Analyses of PC species were carried out in the positive ion mode using precursor scan of 184 with a collision energy of 33 eV to detect the molecular species as the [M + H]^+^ ions. PE species were detected as the [M − H]^−^ ions in the negative ion mode using precursor ion scan of 196 with a collision energy of 50 eV. Quantitation of each individual PC and PE species was compared with internal standards, and results were normalized to the total protein content of the input.

### Isolation of synaptic membranes

To isolate light membrane fractions enriched for synaptic components, dissected retinas (two from each animal, combined) were lysed in 500 μl of high pH buffer containing 500 mM sodium carbonate (pH 11.0) and protease inhibitors (Roche), incubated on ice for 30 min, and homogenized by sonication at 25% amplitude for 20 s with a 50% duty cycle. Homogenates were adjusted to 800 μl of 45% sucrose and placed under sucrose layers of 5% (450 μl) and 35% (1 ml). After centrifugation at 39,000 rpm in an SW-41 rotor (Beckman) at 4 °C for 16 h, 200-μl fractions were sequentially collected from top to bottom. Fractions were analyzed by SDS-PAGE and immunoblotting. The first five fractions were combined as the light membrane component, and the next five fractions were considered the heavy membrane component. Combined fractions were subjected to Folch total lipid extraction and analyzed by electrospray ionization mass spectrometry as described previously.

### Statistics

In line graphs, data are expressed as mean ± SEM. In box-and-whisker plots, data are expressed as median, with the box showing the limits of the interquartile range, and whiskers representing maxima and minima. For experiments with two groups in the independent variable, analyses were performed using two-tailed *t* tests without post hoc correction. For experiments with more than two groups in the independent variable, one-way ANOVA with Bonferroni correction was used when only one dependent variable was present, and two-way ANOVA with Bonferroni post tests was used when two or more dependent variables were present. All calculations were performed using GraphPad Prism 6.0 software (GraphPad Software, Inc). In all experiments, ∗ indicates *p* < 0.05, ∗∗*p* < 0.01, ∗∗∗*p* < 0.001, and ∗∗∗∗*p* < 0.0001.

### Study approval

Protocols followed the Association for Research in Vision and Ophthalmology Statement for the Use of Animals and were approved by Washington University.

## Data availability

All datasets included in this article, including synaptic membrane fatty acid mass spectrometry profiles, will be shared upon request. Please address all inquiries to Rithwick Rajagopal.

## Conflict of interest

The authors declare that they have no conflicts of interest with the contents of this article.

## References

[1] Blinder K.J., Dugel P.U., Chen S., Jumper J.M., Walt J.G., Hollander D.A., Scott L.C. (2017). Anti-VEGF treatment of diabetic macular edema in clinical practice: Effectiveness and patterns of use (ECHO study report 1). Clin. Ophthalmol..

[2] Busik J.V. (2021). Lipid metabolism dysregulation in diabetic retinopathy. J. Lipid Res..

[3] Zheng W., Mast N., Saadane A., Pikuleva I.A. (2015). Pathways of cholesterol homeostasis in mouse retina responsive to dietary and pharmacologic treatments. J. Lipid Res..

[4] Opreanu M., Tikhonenko M., Bozack S., Lydic T.A., Reid G.E., McSorley K.M., Sochacki A., Perez G.I., Esselman W.J., Kern T., Kolesnick R., Grant M.B., Busik J.V. (2011). The unconventional role of acid sphingomyelinase in regulation of retinal microangiopathy in diabetic human and animal models. Diabetes.

[5] Chakravarthy H., Navitskaya S., O'Reilly S., Gallimore J., Mize H., Beli E., Wang Q., Kady N., Huang C., Blanchard G.J., Grant M.B., Busik J.V. (2016). Role of acid sphingomyelinase in shifting the balance between proinflammatory and reparative bone marrow cells in diabetic retinopathy. Stem Cells.

[6] Agbaga M.P., Mandal M.N., Anderson R.E. (2010). Retinal very long-chain PUFAs: New insights from studies on ELOVL4 protein. J. Lipid Res..

[7] Zemski Berry K.A., Gordon W.C., Murphy R.C., Bazan N.G. (2014). Spatial organization of lipids in the human retina and optic nerve by MALDI imaging mass spectrometry. J. Lipid Res..

[8] Bennett L.D., Brush R.S., Chan M., Lydic T.A., Reese K., Reid G.E., Busik J.V., Elliott M.H., Anderson R.E. (2014). Effect of reduced retinal VLC-PUFA on rod and cone photoreceptors. Invest. Ophthalmol. Vis. Sci..

[9] Tikhonenko M., Lydic T.A., Wang Y., Chen W., Opreanu M., Sochacki A., McSorley K.M., Renis R.L., Kern T., Jump D.B., Reid G.E., Busik J.V. (2010). Remodeling of retinal fatty acids in an animal model of diabetes: A decrease in long-chain polyunsaturated fatty acids is associated with a decrease in fatty acid elongases Elovl2 and Elovl4. Diabetes.

[10] Tikhonenko M., Lydic T.A., Opreanu M., Li Calzi S., Bozack S., McSorley K.M., Sochacki A.L., Faber M.S., Hazra S., Duclos S., Guberski D., Reid G.E., Grant M.B., Busik J.V. (2013). N-3 polyunsaturated Fatty acids prevent diabetic retinopathy by inhibition of retinal vascular damage and enhanced endothelial progenitor cell reparative function. PLoS One.

[11] Sala-Vila A., Diaz-Lopez A., Valls-Pedret C., Cofan M., Garcia-Layana A., Lamuela-Raventos R.M., Castaner O., Zanon-Moreno V., Martinez-Gonzalez M.A., Toledo E., Basora J., Salas-Salvado J., Corella D., Gomez-Gracia E., Fiol M. (2016). Dietary marine omega-3 fatty acids and incident sight-threatening retinopathy in middle-aged and older individuals with type 2 diabetes: Prospective investigation from the PREDIMED trial. JAMA Ophthalmol..

[12] Majidi S.P., Rajagopal R. (2020). Photoreceptor responses to light in the pathogenesis of diabetic retinopathy. Vis. Neurosci..

[13] Shindou H., Koso H., Sasaki J., Nakanishi H., Sagara H., Nakagawa K.M., Takahashi Y., Hishikawa D., Iizuka-Hishikawa Y., Tokumasu F., Noguchi H., Watanabe S., Sasaki T., Shimizu T. (2017). Docosahexaenoic acid preserves visual function by maintaining correct disc morphology in retinal photoreceptor cells. J. Biol. Chem..

[14] Kady N.M., Liu X., Lydic T.A., Syed M.H., Navitskaya S., Wang Q., Hammer S.S., O'Reilly S., Huang C., Seregin S.S., Amalfitano A., Chiodo V.A., Boye S.L., Hauswirth W.W., Antonetti D.A. (2018). ELOVL4-mediated production of very long-chain ceramides stabilizes tight junctions and prevents diabetes-induced retinal vascular permeability. Diabetes.

[15] Semenkovich C.F. (1997). Regulation of fatty acid synthase (FAS). Prog. Lipid Res..

[16] Rajagopal R., Zhang S., Wei X., Doggett T., Adak S., Enright J., Shah V., Ling G., Chen S., Yoshino J., Hsu F.F., Semenkovich C.F. (2018). Retinal de novo lipogenesis coordinates neurotrophic signaling to maintain vision. JCI Insight.

[17] Razani B., Zhang H., Schulze P.C., Schilling J.D., Verbsky J., Lodhi I.J., Topkara V.K., Feng C., Coleman T., Kovacs A., Kelly D.P., Saffitz J.E., Dorn G.W., Nichols C.G., Semenkovich C.F. (2011). Fatty acid synthase modulates homeostatic responses to myocardial stress. J. Biol. Chem..

[18] Chakravarthy M.V., Pan Z., Zhu Y., Tordjman K., Schneider J.G., Coleman T., Turk J., Semenkovich C.F. (2005). “New” hepatic fat activates PPARalpha to maintain glucose, lipid, and cholesterol homeostasis. Cell Metab..

[19] Wei X., Song H., Yin L., Rizzo M.G., Sidhu R., Covey D.F., Ory D.S., Semenkovich C.F. (2016). Fatty acid synthesis configures the plasma membrane for inflammation in diabetes. Nature.

[20] Funai K., Song H., Yin L., Lodhi I.J., Wei X., Yoshino J., Coleman T., Semenkovich C.F. (2013). Muscle lipogenesis balances insulin sensitivity and strength through calcium signaling. J. Clin. Invest..

[21] Bogdanov P., Corraliza L., Villena J.A., Carvalho A.R., Garcia-Arumi J., Ramos D., Ruberte J., Simo R., Hernandez C. (2014). The db/db mouse: A useful model for the study of diabetic retinal neurodegeneration. PLoS One.

[22] Feit-Leichman R.A., Kinouchi R., Takeda M., Fan Z., Mohr S., Kern T.S., Chen D.F. (2005). Vascular damage in a mouse model of diabetic retinopathy: Relation to neuronal and glial changes. Invest. Ophthalmol. Vis. Sci..

[23] Rajagopal R., Bligard G.W., Zhang S., Yin L., Lukasiewicz P., Semenkovich C.F. (2016). Functional deficits precede structural lesions in mice with high-fat diet-induced diabetic retinopathy. Diabetes.

[24] Wakil S.J., Abu-Elheiga L.A. (2009). Fatty acid metabolism: Target for metabolic syndrome. J. Lipid Res..

[25] Li S., Chen D., Sauve Y., McCandless J., Chen Y.J., Chen C.K. (2005). Rhodopsin-iCre transgenic mouse line for Cre-mediated rod-specific gene targeting. Genesis.

[26] Najmabadi H., Hu H., Garshasbi M., Zemojtel T., Abedini S.S., Chen W., Hosseini M., Behjati F., Haas S., Jamali P., Zecha A., Mohseni M., Puttmann L., Vahid L.N., Jensen C. (2011). Deep sequencing reveals 50 novel genes for recessive cognitive disorders. Nature.

[27] Bowers M., Liang T., Gonzalez-Bohorquez D., Zocher S., Jaeger B.N., Kovacs W.J., Rohrl C., Cramb K.M.L., Winterer J., Kruse M., Dimitrieva S., Overall R.W., Wegleiter T., Najmabadi H., Semenkovich C.F. (2020). FASN-dependent lipid metabolism links neurogenic stem/progenitor cell activity to learning and memory deficits. Cell Stem Cell.

[28] Honasoge A., Nudleman E., Smith M., Rajagopal R. (2019). Emerging insights and interventions for diabetic retinopathy. Curr. Diabetes Rep..

[29] de Gooyer T.E., Stevenson K.A., Humphries P., Simpson D.A., Gardiner T.A., Stitt A.W. (2006). Retinopathy is reduced during experimental diabetes in a mouse model of outer retinal degeneration. Invest. Ophthalmol. Vis. Sci..

[30] Liu H., Tang J., Du Y., Lee C.A., Golczak M., Muthusamy A., Antonetti D.A., Veenstra A.A., Amengual J., von Lintig J., Palczewski K., Kern T.S. (2015). Retinylamine benefits early diabetic retinopathy in mice. J. Biol. Chem..

[31] Liu H., Tang J., Du Y., Saadane A., Samuels I., Veenstra A., Kiser J.Z., Palczewski K., Kern T.S. (2019). Transducin1, phototransduction and the development of early diabetic retinopathy. Invest. Ophthalmol. Vis. Sci..

[32] Thebeau C., Zhang S., Kolesnikov A.V., Kefalov V.J., Semenkovich C.F., Rajagopal R. (2020). Light deprivation reduces the severity of experimental diabetic retinopathy. Neurobiol. Dis..

[33] Okawa H., Sampath A.P., Laughlin S.B., Fain G.L. (2008). ATP consumption by mammalian rod photoreceptors in darkness and in light. Curr. Biol..

[34] Pinot M., Vanni S., Pagnotta S., Lacas-Gervais S., Payet L.A., Ferreira T., Gautier R., Goud B., Antonny B., Barelli H. (2014). Lipid cell biology. Polyunsaturated phospholipids facilitate membrane deformation and fission by endocytic proteins. Science.

[35] Chen Y.F., Chen H.Y., Lin C.C., Chen M.S., Chen P.C., Wang I.J. (2012). Retinitis pigmentosa reduces the risk of proliferative diabetic retinopathy: A nationwide population-based cohort study. PLoS One.

[36] Arden G.B. (2001). The absence of diabetic retinopathy in patients with retinitis pigmentosa: Implications for pathophysiology and possible treatment. Br. J. Ophthalmol..

[37] Sternberg P., Landers M.B., Wolbarsht M. (1984). The negative coincidence of retinitis pigmentosa and proliferative diabetic retinopathy. Am. J. Ophthalmol..

[38] Nippert A.R., Biesecker K.R., Newman E.A. (2018). Mechanisms mediating functional hyperemia in the brain. Neuroscientist.

[39] Rumora A.E., LoGrasso G., Hayes J.M., Mendelson F.E., Tabbey M.A., Haidar J.A., Lentz S.I., Feldman E.L. (2019). The divergent roles of dietary saturated and monounsaturated fatty acids on nerve function in murine models of obesity. J. Neurosci..

[40] Metea M.R., Newman E.A. (2006). Glial cells dilate and constrict blood vessels: A mechanism of neurovascular coupling. J. Neurosci..

